# Supplementary figures and images for: Growth-dependent signals drive an increase in early G1 cyclin concentration to link cell cycle entry with cell growth
Source: eLife. 2021 Oct 29;10:e64364. doi: 10.7554/eLife.64364 (PMC8592568; doi:10.7554/eLife.64364)

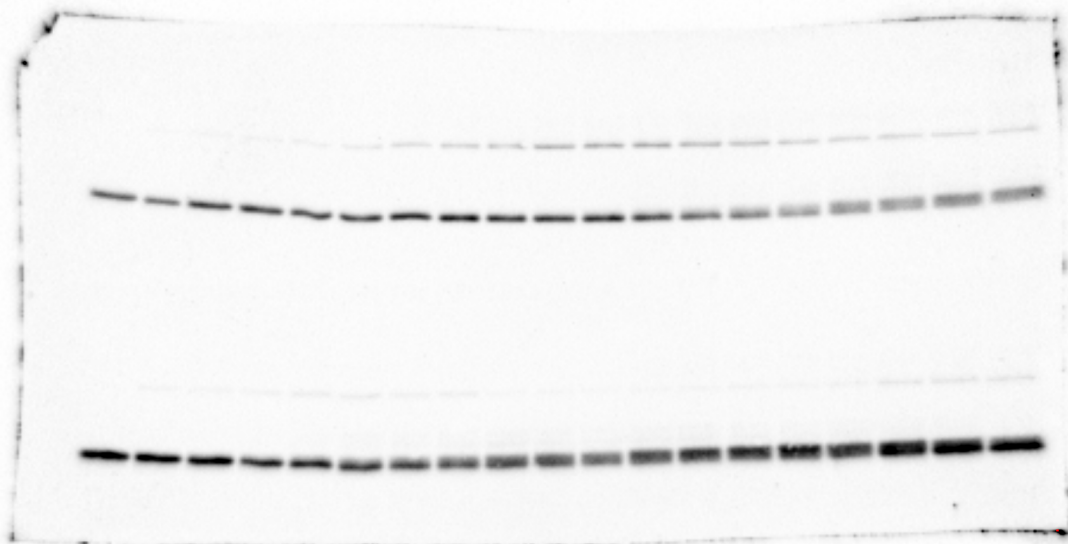

Supplement: Figure 1—source data 1. [file elife-64364-fig1-data1.pdf]

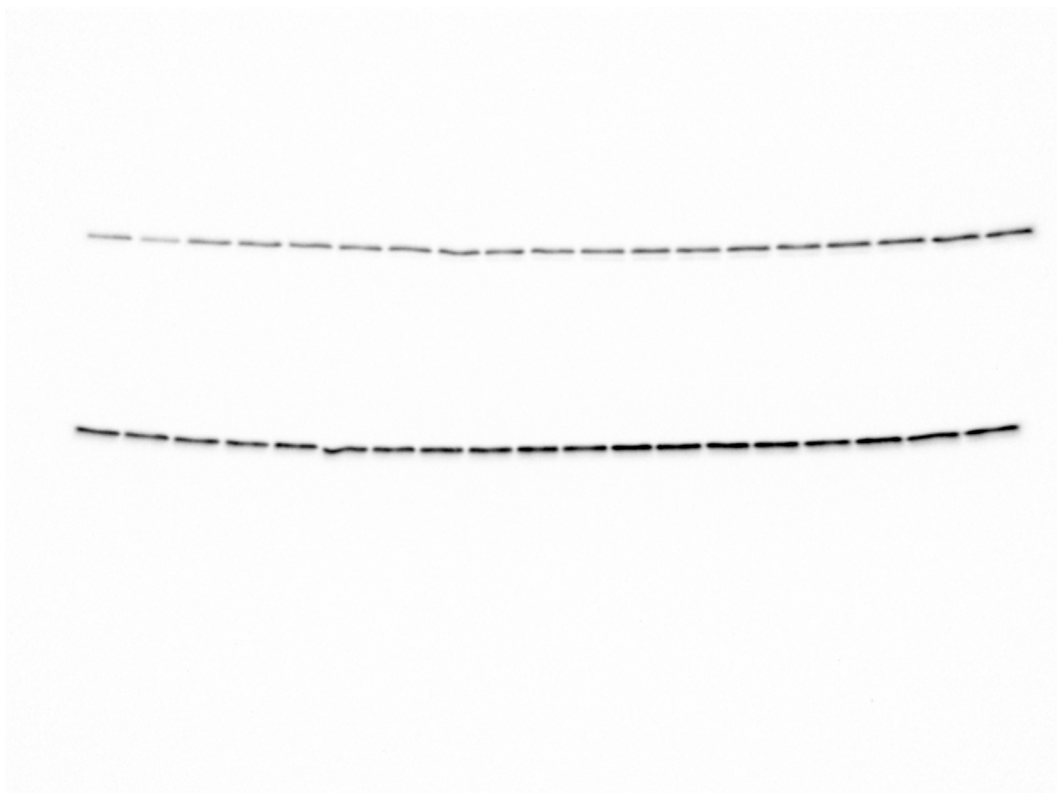

Supplement: Figure 1—source data 2. [file elife-64364-fig1-data2.pdf]

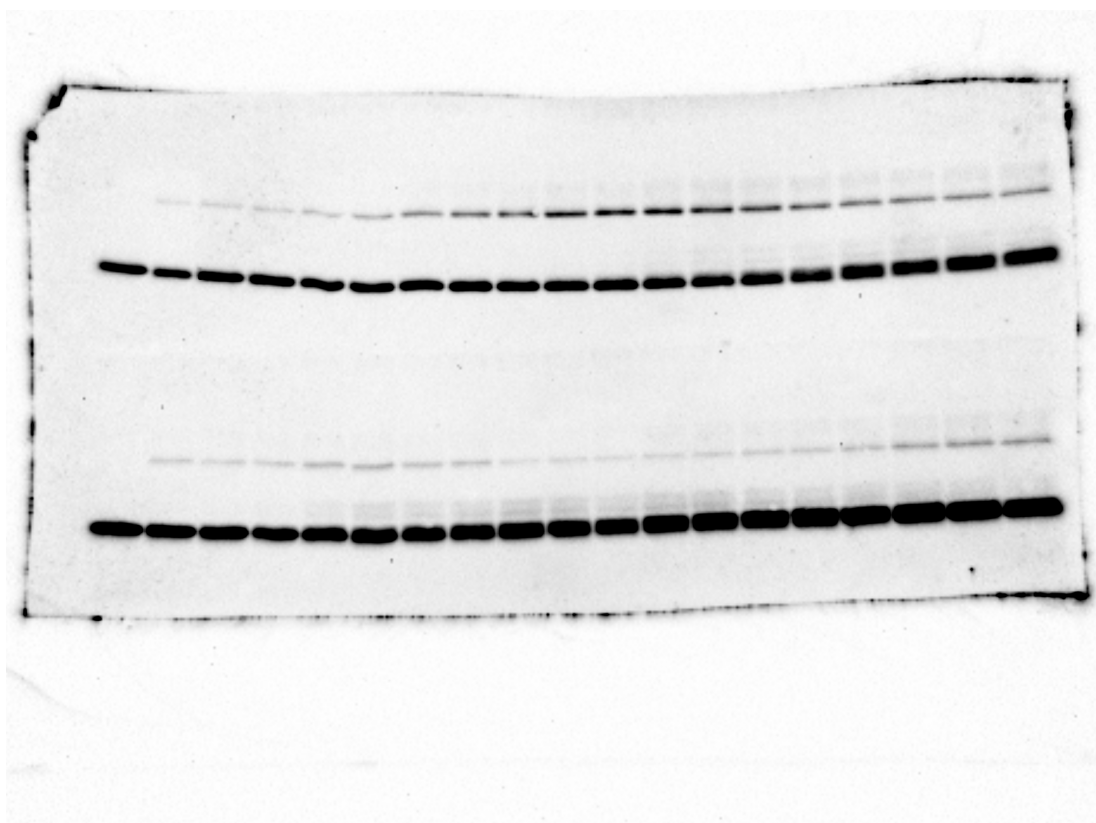

Supplement: Figure 1—figure supplement 1—source data 1. [file elife-64364-fig1-figsupp1-data1.pdf]

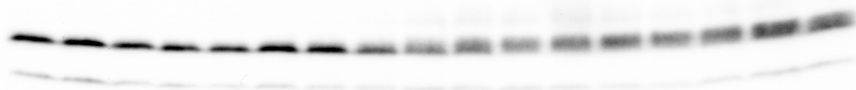

Supplement: Figure 1—figure supplement 2—source data 1. [file elife-64364-fig1-figsupp2-data1.pdf]

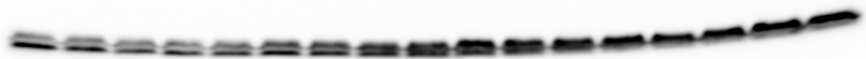

Supplement: Figure 1—figure supplement 2—source data 2. [file elife-64364-fig1-figsupp2-data2.pdf]

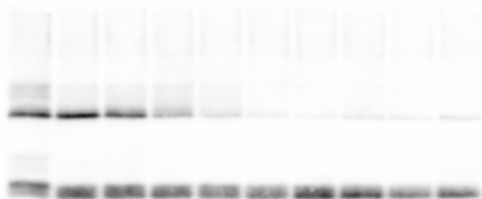

Supplement: Figure 2—source data 1. [file elife-64364-fig2-data1.pdf]

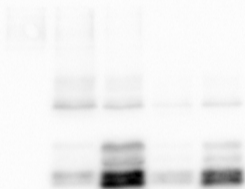

Supplement: Figure 3—figure supplement 1—source data 1. [file elife-64364-fig3-figsupp1-data1.pdf]

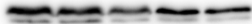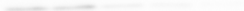

Supplement: Figure 3—figure supplement 1—source data 2. [file elife-64364-fig3-figsupp1-data2.pdf]

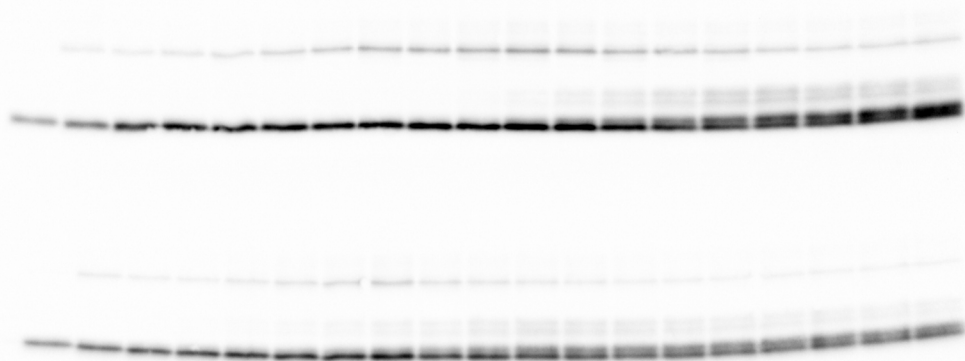

Supplement: Figure 3—figure supplement 1—source data 3. [file elife-64364-fig3-figsupp1-data3.pdf]

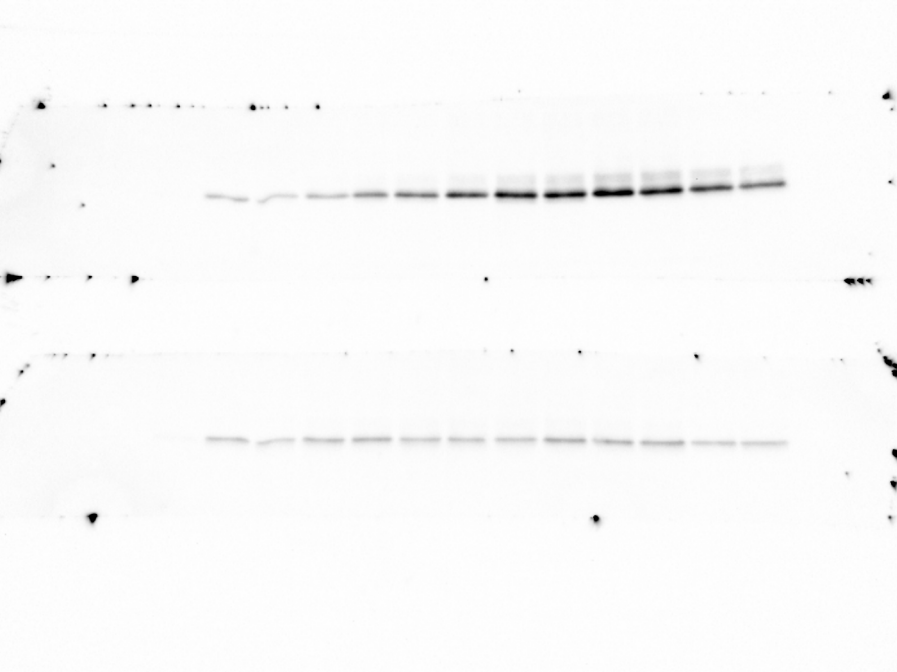

Supplement: Figure 4—source data 1. [file elife-64364-fig4-data1.pdf]

SALT-AID  
ST-17      AUXIN

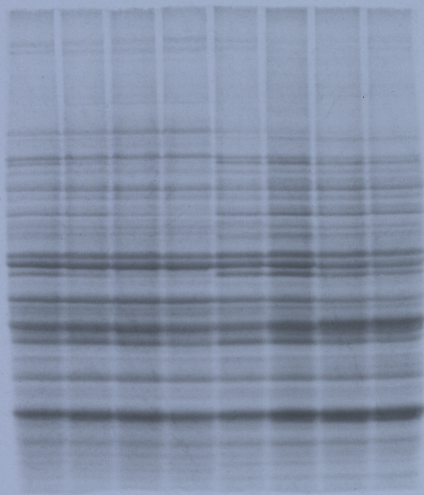

Supplement: Figure 4—source data 3. [file elife-64364-fig4-data3.pdf]

Ex 342  
9/12  
24hr

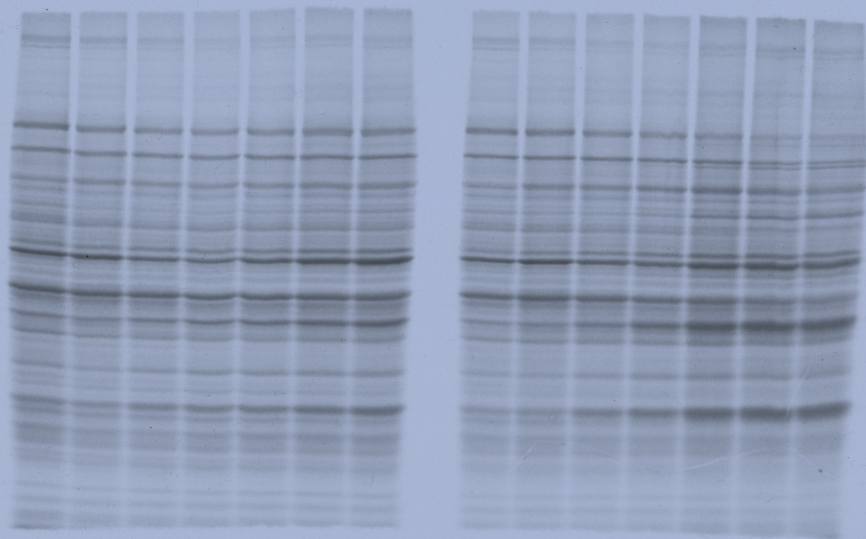

Supplement: Figure 4—figure supplement 1—source data 1. [file elife-64364-fig4-figsupp1-data1.pdf]

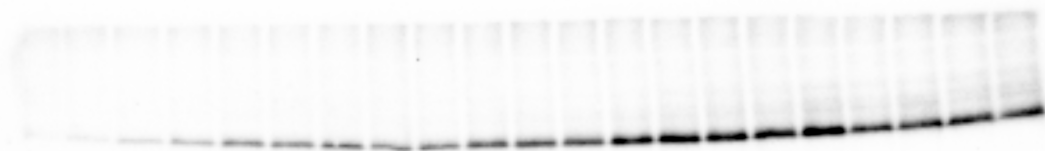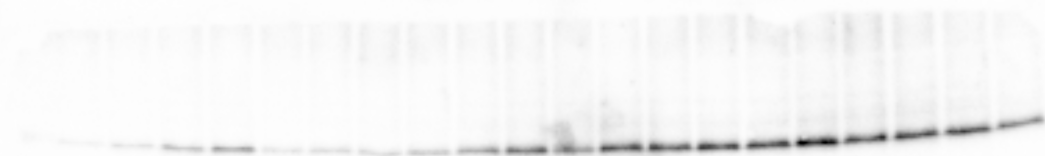

Supplement: Figure 5—source data 1. [file elife-64364-fig5-data1.pdf]

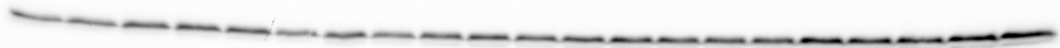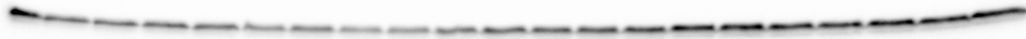

Supplement: Figure 5—source data 2. [file elife-64364-fig5-data2.pdf]

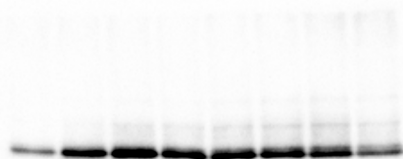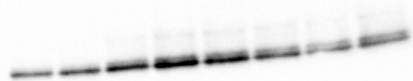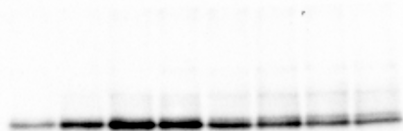

Supplement: Figure 5—figure supplement 1—source data 1. [file elife-64364-fig5-figsupp1-data1.pdf]

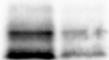

Supplement: Figure 6—source data 3. [file elife-64364-fig6-data3.pdf]

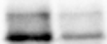

Supplement: Figure 6—source data 6. [file elife-64364-fig6-data6.pdf]

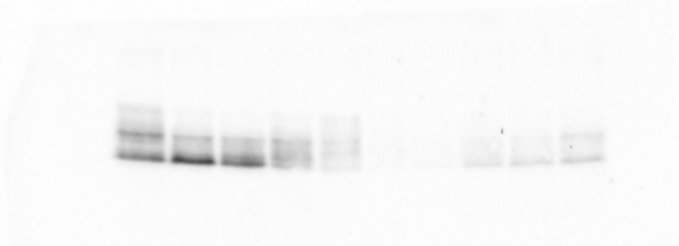

Supplement: Figure 6—source data 7. [file elife-64364-fig6-data7.pdf]

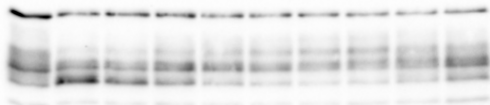

Supplement: Figure 6—source data 8. [file elife-64364-fig6-data8.pdf]

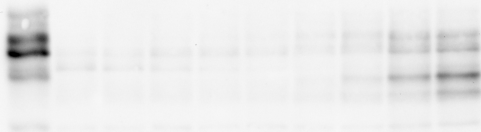

Supplement: Figure 6—source data 9. [file elife-64364-fig6-data9.pdf]

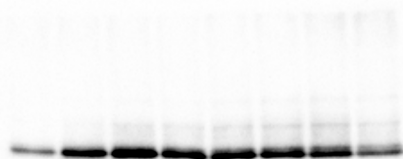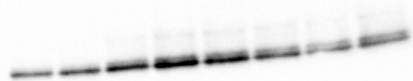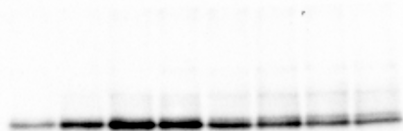

Supplement: Figure 6—figure supplement 1—source data 1. [file elife-64364-fig6-figsupp1-data1.pdf]

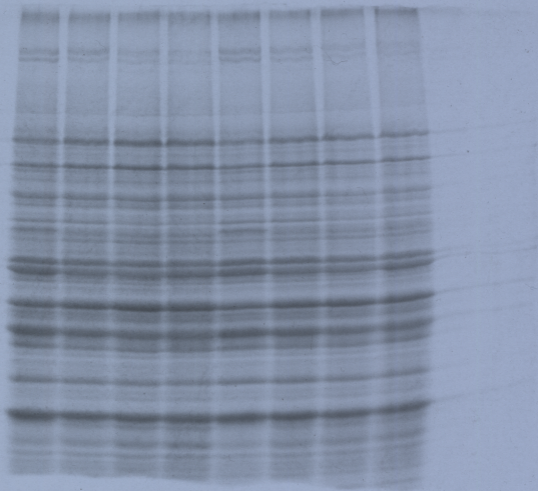

Supplement: Figure 6—figure supplement 1—source data 3. [file elife-64364-fig6-figsupp1-data3.pdf]

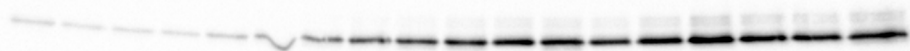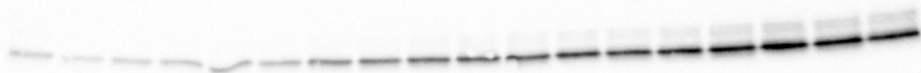

Supplement: Figure 7—source data 1. [file elife-64364-fig7-data1.pdf]

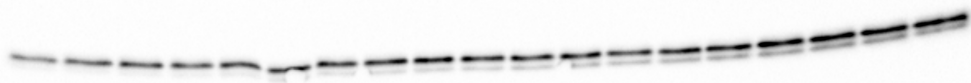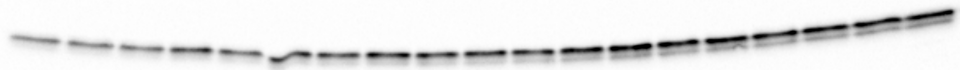

Supplement: Figure 7—source data 2. [file elife-64364-fig7-data2.pdf]

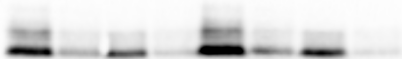

Supplement: Figure 7—source data 3. [file elife-64364-fig7-data3.pdf]

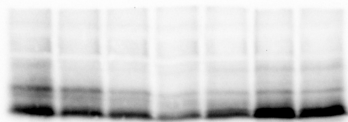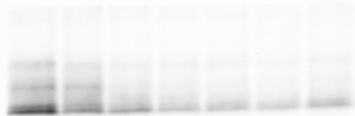

Supplement: Figure 7—source data 5. [file elife-64364-fig7-data5.pdf]
