# Supplementary material for: Growth-dependent signals drive an increase in early G1 cyclin concentration to link cell cycle entry with cell growth
Source: eLife. 2021 Oct 29;10:e64364. doi: 10.7554/eLife.64364 (PMC8592568; doi:10.7554/eLife.64364)

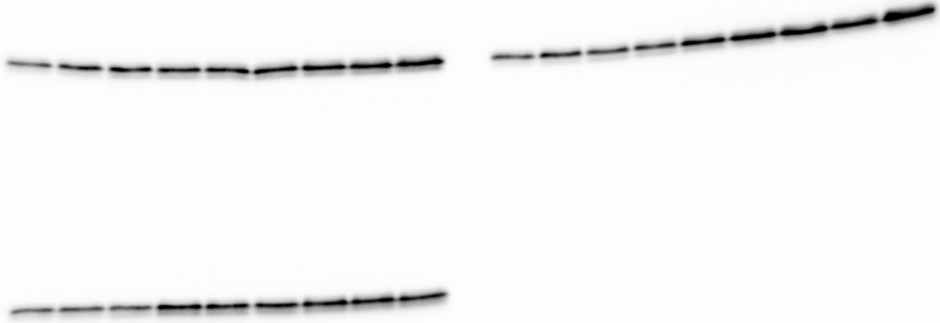

Supplement: Figure 6—figure supplement 1—source data 2. [file elife-64364-fig6-figsupp1-data2.pdf]
