# Supplementary material for: Growth-dependent signals drive an increase in early G1 cyclin concentration to link cell cycle entry with cell growth
Source: eLife. 2021 Oct 29;10:e64364. doi: 10.7554/eLife.64364 (PMC8592568; doi:10.7554/eLife.64364)

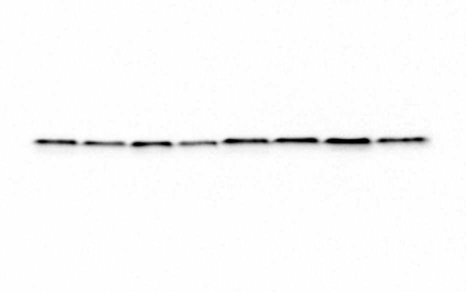

Supplement: Figure 7—source data 4. [file elife-64364-fig7-data4.pdf]
